# Protein-Protein Interaction Analysis Highlights Additional Loci of Interest for Multiple Sclerosis

**DOI:** 10.1371/journal.pone.0046730

**Published:** 2012-10-18

**Authors:** Giammario Ragnedda, Giulio Disanto, Gavin Giovannoni, George C. Ebers, Stefano Sotgiu, Sreeram V. Ramagopalan

**Affiliations:** 1 Wellcome Trust Centre for Human Genetics, University of Oxford, Oxford, United Kingdom; 2 Nuffield Department of Clinical Neurosciences (Clinical Neurology), University of Oxford, The West Wing, John Radcliffe Hospital, Oxford, United Kingdom; 3 Division of Neurology, Department of Clinical and Experimental Medicine, University of Sassari, Sassari, Italy; 4 Blizard Institute, Queen Mary University of London, Barts and The London School of Medicine and Dentistry, London, United Kingdom; 5 Division of Paediatric Neuropsychiatry, Department of Clinical and Experimental Medicine, University of Sassari, Sassari, Italy; 6 London School of Hygiene and Tropical Medicine, London, United Kingdom; City of Hope National Medical Center and Beckman Research Institute, United States of America

## Abstract

Genetic factors play an important role in determining the risk of multiple sclerosis (MS). The strongest genetic association in MS is located within the major histocompatibility complex class II region (MHC), but more than 50 MS loci of modest effect located outside the MHC have now been identified. However, the relative candidate genes that underlie these associations and their functions are largely unknown. We conducted a protein-protein interaction (PPI) analysis of gene products coded in loci recently reported to be MS associated at the genome-wide significance level and in loci suggestive of MS association. Our aim was to identify which suggestive regions are more likely to be truly associated, which genes are mostly implicated in the PPI network and their expression profile. From three recent independent association studies, SNPs were considered and divided into significant and suggestive depending on the strength of the statistical association. Using the Disease Association Protein-Protein Link Evaluator tool we found that direct interactions among genetic products were significantly higher than expected by chance when considering both significant regions alone (*p<*0.0002) and significant plus suggestive (*p<*0.007). The number of genes involved in the network was 43. Of these, 23 were located within suggestive regions and many of them directly interacted with proteins coded within significant regions. These included genes such as *SYK*, *IL-6*, *CSF2RB*, *FCLR3*, *EIF4EBP2* and *CHST12*. Using the gene portal BioGPS, we tested the expression of these genes in 24 different tissues and found the highest values among immune-related cells as compared to non-immune tissues (*p*<0.001). A gene ontology analysis confirmed the immune-related functions of these genes. In conclusion, loci currently suggestive of MS association interact with and have similar expression profiles and function as those significantly associated, highlighting the fact that more common variants remain to be found to be associated to MS.

## Introduction

Multiple Sclerosis (MS) is the most common inflammatory disease of central nervous system (CNS) which affects young adults [1]. It is widely acknowledged that genetic factors play an important role in determining the risk of MS [2]. Several epidemiological studies demonstrated an increased frequency of MS among biological relatives of affected individuals [3], [4]. Family based and association studies have shown that the strongest genetic association in MS is located within the major histocompatibility complex (MHC) class II region [5]. In particular the HLA-DRB1*1501 allele confers an approximate odds ratio of 3 [6]. However, during the last few years Genome Wide Association Studies (GWAS) have identified many other MS associated loci of modest effect located outside the MHC (now more than 50) [7]–[11].

Despite the recent advances in the understanding of the genetic architecture of MS, several questions remain to be answered. For example, due to stringent correction criteria many genetic variants fail to reach genome-wide significance but can still be considered as suggestive of genetic association. Furthermore, once a SNP is found to be associated with a particular disease, the relative candidate gene (or genes) that mediate such association is usually unknown.

Analysis of protein-protein interaction (PPI) networks is being increasingly recognized as an important tool to characterize the underlying biology of genes associated to complex diseases, in particular immune-mediated ones [12], [13]. It is logical to hypothesize that those genes which are truly associated with the same trait will be involved in similar biological processes. For example, Rossin et al. found that proteins encoded in genomic regions associated to rheumatoid Arthritis and Crohn's disease physically interact more than what would be expected by chance and that the genes encoding these proteins are highly expressed in immune tissues [12]. Studying such PPI interactions can ultimately elucidate which suggestive regions are more likely to be truly associated and greatly aid the identification of those genes that are mediating the GWAS findings.

We conducted a PPI analysis of gene products coded in loci recently reported to be MS associated and suggestive of MS association. Our aim was to identify which suggestive regions are more likely to be truly associated, which genes are mostly implicated in the MS PPI network, their expression profiles and functions.

## Methods

Three recent independent association studies were considered for our analysis [14]–[16]. In Sawcer et al. and Patsopoulos et al., SNPs were divided into significant and suggestive depending on the strength of the statistical association [14], [15]. From Sawcer et al we defined as suggestive those SNPs with *p* values in the discovery phase of less than 1×10^−4^ and significant those that either were replication of previous GWAS findings or had a replication *p<*0.05 and a *p*-combined*<*5×10^−7^ [14]. In Patsopoulos et al., significant SNPs were defined as either those with *p*-value<5×10^−8^ or replication of previously identified associated SNPs. Suggestive SNPs were those with *p*-values between 5×10^−8^ and 1×10^−6^ [15]. We also included in the analyses the top 82 SNPs (with a log *p* value*>*4.91) from Wang et al [16]. All SNPs from this study were considered as suggestive, because the study was not designed to meet currently accepted criteria for genome wide significance. After removing duplicate SNPs, 67 significant and 133 suggestive SNPs were obtained.

Protein-to-protein interaction assessment was conducted using the Disease Association Protein-Protein Link Evaluator (DAPPLE) tool [12]. This bioinformatics tool is able to investigate physical interactions among gene products encoded within certain genomic regions by the creation of a PPI network. Interactions are extracted from the database “InWeb” that combines data from a variety of public PPI sources including MINT, BIND, IntAct and KEGG and defines high confidence interactions as those seen in multiple independent experiments. The region around a given SNP is extended to the genomic interval defined by SNPs in moderate linkage disequilibrium (r∧2> = 0.5) and then to the nearest recombination hotspots [12]. Connections can be direct (two proteins are physically linked to each other) and indirect (interaction is mediated by a common interactor). The extent of the PPI network are assessed using the following parameters: the number of direct interactions between proteins from different loci, the mean associated protein direct and indirect connectivities (the mean number of distinct loci a protein is directly or indirectly connected to) and the mean common interactor connectivity (average number of proteins in separate loci bound by common interactors) [12]. The non-randomness of the network and the significance of the interaction parameters are tested using a permutation method that compares the original network with thousands of networks created by randomly re-assigning the protein names while keeping the overall structure (size and number of interactions) of the original network. Those genes that participate in the network more than expected by chance are defined as genes to prioritize (corrected *p*<0.05) [12]. Expression data were gathered from BioGPS, an online gene annotation database that reports individual gene expression levels for a number of human tissues and cell types [17]. Analyses were performed using non-parametric tests (Kruskal-Wallis and Mann-Whitney tests). Gene ontology terms were investigated using The **D**atabase for **A**nnotation, **V**isualization and **I**ntegrated **D**iscovery (**DAVID**) v6.7, an online tool that is able to identify the functional categories and biological processes which are most represented within a list of genes [18], [19].

## Results

### Dapple analysis of significant SNPs

**Figure 1:**
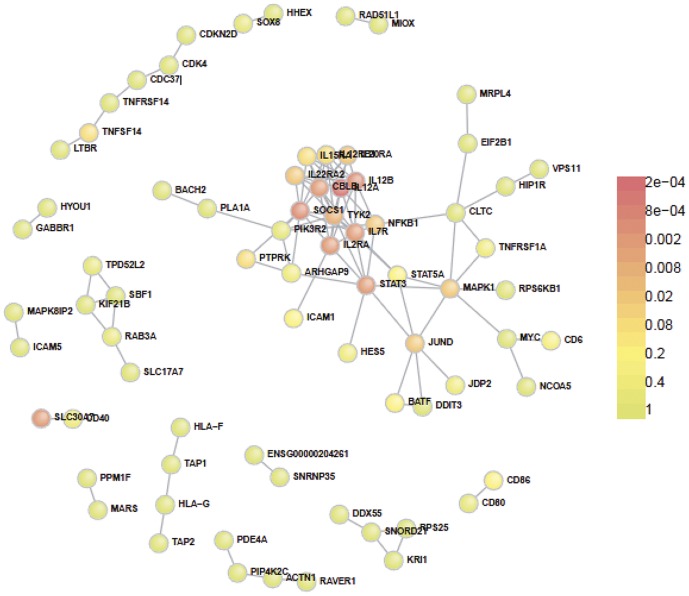
Direct connections among gene products from MS significant regions. Colours indicate significance of participation in the PPI network.

Our first aim was to assess the extent of PPI interactions among genes located within genomic regions with definite association with MS susceptibility. We therefore submitted into DAPPLE the 67 SNPs with genome-wide significant association with MS risk. There were a total of 75 proteins participating in the direct network with 104 direct interactions (expected direct interactions = 61, *p*<0.0002) (Table 1, Figure 1 and Table S1). The mean associated protein direct connectivity was 2.7 (expected = 1.7, *p*<0.0002). The mean associated protein indirect connectivity was 52.2 (expected = 43.8, *p = *0.04) and the mean common interactor connectivity was 4.5. (expected = 3.9, *p = *0.0002). The total number of genes implicated in the network was 215 (Table S1). The total number of genes that had more connections than expected by chance (genes to prioritize) was 22 and included previously shown putative candidate genes such as IL*-12A*, *SOCS-1*, *CBLB*, *MALT-1*, *IL-22RA*, *MAPK-1* and *IL-7R*.

**Table 1:** Summary of DAPPLE analysis of significant and significant plus suggestive SNPs.

	Significant	Significance	Significant + suggestive	Significance
**Number of proteins in the network**	75	-	189	-
**Direct interactions**	104	*p<*0.0002	281	*p<*0.007
**MAPDC** *	2.7	*p<*0.0002	2.9	*p = *0.0008
**MAPIC** **	52.2	*p = *0.04	93	*p = *0.34
**Mean CI connectivity** ***	4.5	*p = *0.0002	5.05	*p = *0.05
**Genes to prioritize**	39	-	22	-

* Mean associated protein direct connectivity;

** Mean associated protein indirect connectivity;

*** Mean Common Interactor connectivity.

### Dapple analysis of significant plus suggestive SNPs

**Figure 2:**
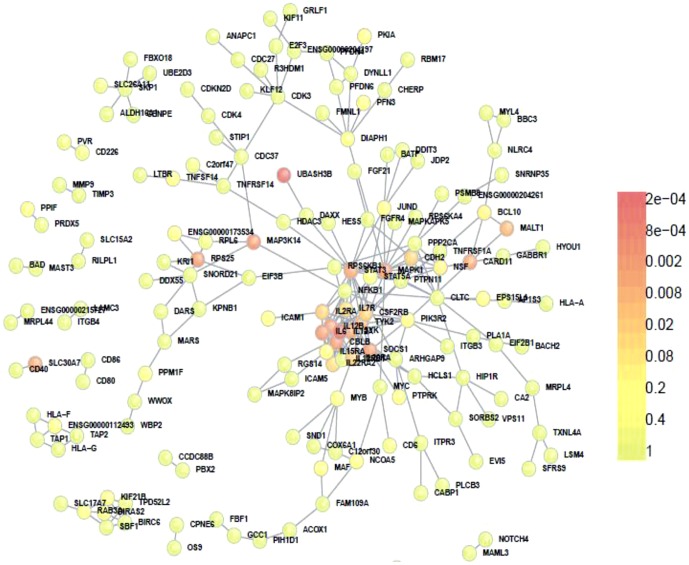
Direct connections among gene products from MS significant plus suggestive regions. Colours indicate significance of participation in the PPI network.

When suggestive SNPs were included in the analysis, the number of proteins participating in the network and that of direct interactions increased from 75 to 189 and from 104 to 281 respectively (expected direct interactions = 242, *p*<0.007) (Table 1, Figure 2 and Table S2). The mean associated protein direct connectivity was also higher than expected (observed = 2.9, expected = 2.4, *p = *0.0008). The mean associated protein indirect connectivity was 93 (expected = 91, *p = *0.34). The mean common interactor connectivity was 5.05 (expected = 4.8, *p = *0.05). The total number of genes analyzed was 445 (Table S2), while genes to prioritize were 43 of which 23 were located within suggestive regions. These included genes such as *SYK*, *IL-6*, *CSF2RB*, *FCLR3*, *EIF4EBP2* and *CHST12* (Table 2).

**Table 2:** List of candidate genes (genes to prioritize) obtained from DAPPLE analysis of significant plus suggestive SNPs.

	SIGNIFICANT			SUGGESTIVE	
GENE	SNP	STUDY	GENE	SNP	STUDY
*BCL9L*	rs630923	14	*ARAP3*	rs2302103	14
*CARD11*	rs11581062	14	*CHST12*	rs6952809	14
*CBLB*	rs2028597	14	*CSF2RB*	rs2072711	14
*IL12A*	rs2243123	14	*FCRL3*	rs3761959	14
*IL12B*	rs2546890	14,15	*MAP3K14*	rs4792814	14
*IL20RA*	rs17066096	14	*NDFIP1*	rs1062158	14
*IL22RA2*	rs17066096	14	*SLC30A7*	rs12048904	14
*IL2RA*	rs3118470	14	*SYK*	rs290986	14
*IL7R*	rs6897932	14,15	*UBASH3B*	rs7941030	14
*MALT1*	rs7238078	14	*IQCB1*	rs2681424	15
*MAPK1*	rs2283792	14	*ANGPT2*	rs2515585	16
*RPS25*	rs630923	14	*C12orf51*	rs11065987	16
*SOCS1*	rs7200786	14	*CDH2*	rs528438	16
*SP110*	rs10201872	14	*CUX2*	rs11065987	16
*SP140*	rs10201872	14	*EIF4EBP2*	rs10762363	16
*STAT3*	rs9891119	14	*ENSG00000205175*	rs1611715	16
*TMEM87B*	rs17174870	14	*ENSG00000204600*	rs434496	16
*TYK2*	rs8112449	14	*ENSG00000205173*	rs434446	16
*YPEL2*	rs180515	14	*IL6*	rs10244467	16
*C12orf65*	rs1790100	15	*RBM45*	rs10203141	16
			*SLC30A6*	rs13029809	16
			*TRAFD1*	rs11065987	16
			*Wdr23(DCAF11)*	rs10146906	16

### Tissue-specific expression and gene ontology terms of candidate genes

**Figure 3:**
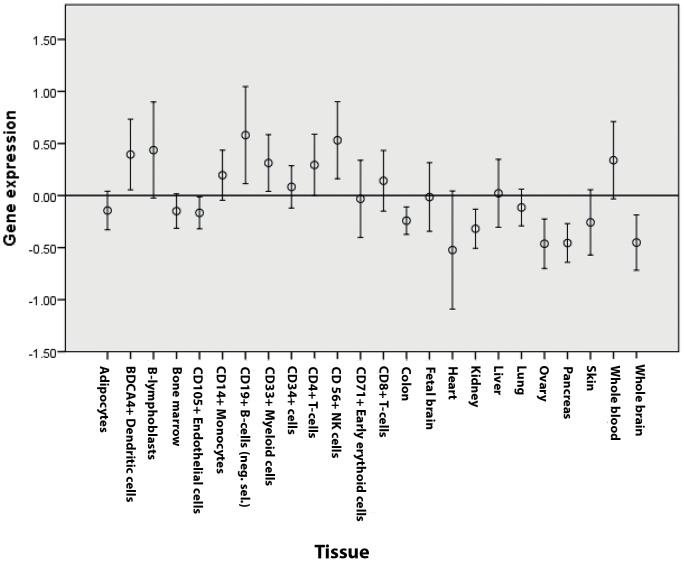
Expression values of candidate genes (genes to prioritize) in all 23 tissues and cell types tested.

**Figure 4:**
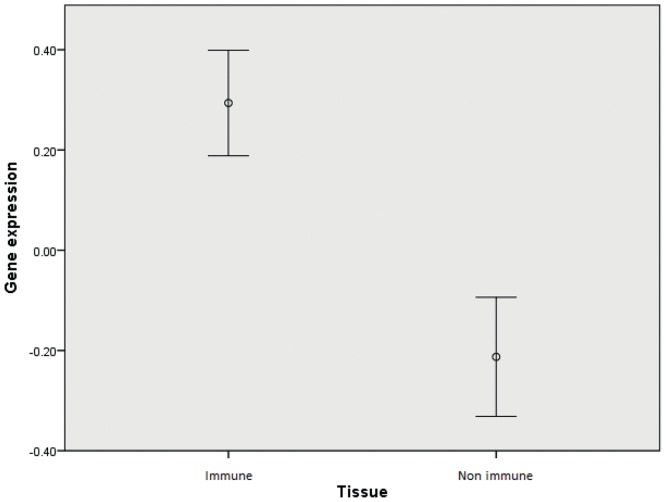
Expression values of candidate genes (genes to prioritize) in immune and non immune tissues.

In order to further investigate the nature of our findings we assessed in which tissues these genes were mostly expressed. We used the gene portal BioGPS which contains gene expression data on a variety of human tissues and cell types [17]. For our analysis we considered 10 immune cell types and 14 non-immune tissues. We submitted the full list of candidate genes (n = 43) obtained from the significant plus suggestive DAPPLE analysis and for each gene we obtained a different genetic expression value in every tissue or cell type tested. Because of different background characteristics between each probe set, a direct comparison of expression across different genes was not possible. Therefore, we decided to standardize the expression values of each single gene across different tissues and used the obtained z-values for all subsequent analyses. Figure 3 shows the standardized expression values in the 24 tissues and cell types tested. Expression appeared particularly high in whole blood as well as in most of immune-related cell types (in particular B-cells, plasmacytoid dendritic cells (pDCs), natural killer (NK) cells, CD4+ and CD8+ T cells). An independent-sample Kruskal-Wallis test confirmed that gene expression was significantly different across tissues (*p*<0.001). When tissues were divided into immune and non-immune, expression was substantially different between the two groups (*p*<0.001) (Figure 4). When compared to average expression across tissues, candidate genes were significantly overexpressed in B-lymphoblasts, pDCs, monocytes, B cells, NK cells, CD4+ T cells (*p*<0.001), CD34+ hematopoietic cells (*p = *0.001) and CD8+ T cells (*p = *0.003). Expression patterns were similar for significantly and suggestively associated loci.

We further confirmed the immunological nature of these candidate genes using **DAVID** [18], [19], a bioinformatics tool that is able to identify the biological processes in which a group of genes are involved. Candidate genes were significantly enriched for immune related processes such as regulation of leukocyte activation (*p = *3.10×10^−8^), regulation of T cell proliferation (*p = *3.25×10^−8^), positive regulation of immune system processes (*p = *7.7×10^−7^), regulation of protein kinase cascade (*p = *5.46×10^−4^) and regulation of cytokine production (*p = *0.001459) (see Table S3 for the full list). GO enrichment was similar for significantly and suggestively associated loci.

## Discussion

We showed that genetic products coded in loci strongly associated with MS risk substantially interact with each other. Both direct and indirect interactions were significantly higher than what would be expected by chance only. When the PPI analysis was extended to suggestive SNPs, we found an increased number of total proteins participating in the network and direct interactions (Figure 1 and 2). The only parameter that did not reach significance was the number of indirect interactions. This finding could be explained by the possible lack of real MS association among several suggestive SNPs.

However, including suggestive SNPs in the PPI analysis increased the number of genes to prioritize from 22 to 43. Interestingly, more than half of these genes (n = 23) were located within suggestive regions and many of them directly interacted with proteins coded within significant regions (e.g. *CSF2RB-CBLB*, *IL6-IL2RA*, *MAPK3K14-NFKB1*, *SYK*-*STAT3*, see Table S2). Taken together the suggestive statistical evidence of genetic association and the functional evidence of protein-protein interaction support the hypothesis that these genes could play an important role in the pathogenesis of MS.

We validated our results looking at tissue specific expression of these candidate genes. Using the BioGPS database we were able to show that the suggestively associated genes identified by DAPPLE were largely and specifically expressed in immune cells as compared to other tissues. A gene ontology analysis also confirmed the immune-related functions of these genes. More generally, these findings provide additional support to the immunological nature of MS [20]. Notably, candidate gene expression was particularly high among CD8+ and CD4+ T cells, B cells, NK cells and pDCs. Interestingly all these cell types have been implicated in the pathogenesis of MS.

Several immune specific genes are located within MS suggestive regions. For example a SNP located near the gene encoding the Spleen Tyrosine Kinase (*SYK*) was found suggestive of association in Sawcer et al. Notably *SYK* was particularly highly expressed in B-cells, DCs, monocytes, CD33+ myeloid cells and NK cells. This protein has a central role in adaptive immune receptor signalling by phosphorylation of the immunoreceptor tyrosine-based activation motifs (ITAMs) [21]. SYK mediated ITAMs phosphorylation determines activation of signalling intermediates such as NF-κB, JNK and PYK2 that ultimately lead to lymphocyte activation [22]. ITAM signals mediated by SYK can also induce expansion of NK cells [23]. Interestingly, the SYK-inhibitor R788 (fostamatinib) has beneficial effects in patients affected by RA, when compared to placebo [24].

*CSF2RB* is another gene particularly highly expressed in B-cells, DCs, monocytes, CD33+ myeloid cells and NK cells. It codes for the β-subunit (βc) of the granulocyte-macrophage colony-stimulating factor (GM-CSF), IL-3 and IL-5 receptors that are expressed by peripheral leucocytes and blood DCs [25]. This gene appears to play an important role in allergic inflammation [26]. Interestingly, associations between *CSF2RB* and schizophrenia [27] and bipolar disorder [28] have been recently found.

*EIF4EBP2* encodes the Eukaryotic Translation Initiation Factor 4E Binding Protein 2. The members of this family of proteins (4EBPs) can inhibit translation initiation through binding eIF4E [29]. 4EBPs regulate cell proliferation by interaction with mTORC1 pathway [30]. In addiction, *EIF4EBP1* knock-out mice showed a type I IFN over production in pDCs [31]. We found an over-expression of *EIF4BP2* in pDCs, CD4 cells, CD8 cells and NK cells. *CHST12* encodes the carbohydrate (chondroitin 4-O) sulfotransferase 2, a protein located in the membrane of the Golgi apparatus membrane and which is implicated in chondroitin and dermatan sulphate (DS) synthesis in different tissues [32]. DS proteoglycans participate in various biological events such as extracellular matrix assembly, cell adhesion, migration and proliferation [33]. We found high expression of *CHST12* in pDCs, CD4 cells, CD8 cells and NK cells.

To conclude, a number of proteins coded by genes located within MS-associated genomic regions are implicated in the same PPI networks. The extent of this interaction substantially increases when genomic regions with suggestive evidence of association are included in the analysis. This suggests that at least some of these suggestive GWAS hits represent truly associated loci, and thus more common variants remain to be found to be associated to MS. Finally, we further confirmed the immunological nature of MS and show how a single cell type cannot explain the complexity of this disease. Future functional studies should investigate how and in which cell types the suggestive candidate genes are acting. This will improve our knowledge of this complex disease and hopefully provide future strategies of disease prevention and treatment.

## Supporting Information

Table S1 **Direct connections and list of genes from DAPPLE analysis of significant SNPs.**
(XLSX)

Table S2 **Direct connections and list of genes from DAPPLE analysis of significant plus suggestive SNPs.**
(XLSX)

Table S3 **Results** **of DAVID gene ontology.**
(XLSX)

## References

[1] Ramagopalan SV, Dobson R, Meier UC, Giovannoni G (2010) Multiple sclerosis: risk factors, prodromes, and potential causal pathways. Lancet Neurol 9 7:727–39. 20610348 10.1016/S1474-4422(10)70094-6

[2] Dyment DA, Ebers GC, Sadovnick AD (2004) Genetics of multiple sclerosis. Lancet Neurol 3 2:104–10. 14747002 10.1016/s1474-4422(03)00663-x

[3] Ebers GC, Sadovnick AD, Risch NJ (1995) A genetic basis for familial aggregation in multiple sclerosis. Canadian Collaborative Study Group. Nature 377 6545:150–1. 7675080 10.1038/377150a0

[4] Ebers GC, Sadovnick AD, Dyment DA, Yee IM, Willer CJ, et al (2004) Parent-of-origin effect in multiple sclerosis: observations in half-siblings. Lancet 363 9423:1773–4. 15172777 10.1016/S0140-6736(04)16304-6

[5] Dyment DA, Herrera BM, Cader MZ, Willer CJ, Lincoln MR, et al (2005) Complex interactions among MHC haplotypes in multiple sclerosis: susceptibility and resistance. Hum Mol Genet 14 14:2019–26. 15930013 10.1093/hmg/ddi206

[6] Ramagopalan SV, Ebers GC (2009) Multiple sclerosis: major histocompatibility complexity and antigen presentation. Genome Med 1 11:105. 19895714 10.1186/gm105 PMC2808740

[7] Hafler DA, Compston A, Sawcer S, Lander ES, Daly MJ, et al (2007) Risk alleles for multiple sclerosis identified by a genomewide study. N Engl J Med 357 9:851–62. 17660530 10.1056/NEJMoa073493

[8] Baranzini SE, Wang J, Gibson RA, Galwey N, Naegelin Y, et al (2009) Genome-wide association analysis of susceptibility and clinical phenotype in multiple sclerosis. Hum Mol Genet 18 4:767–78. 19010793 10.1093/hmg/ddn388 PMC4334814

[9] Australia and New Zealand Multiple Sclerosis Genetics Consortium (ANZgene) (2009) Genome-wide association study identifies new multiple sclerosis susceptibility loci on chromosomes 12 and 20. Nat Genet 41 7:824–8. 19525955 10.1038/ng.396

[10] De Jager PL, Jia X, Wang J, de Bakker PI, Ottoboni L, et al (2009) Meta-analysis of genome scans and replication identify CD6, IRF8 and TNFRSF1A as new multiple sclerosis susceptibility loci. Nat Genet 41 7:776–82. 19525953 10.1038/ng.401 PMC2757648

[11] Sanna S, Pitzalis M, Zoledziewska M, Zara I, Sidore C, et al (2010) Variants within the immunoregulatory CBLB gene are associated with multiple sclerosis. Nat Genet 42 6:495–7. 20453840 10.1038/ng.584 PMC3786343

[12] Rossin EJ, Lage K, Raychaudhuri S, Xavier RJ, Tatar D, et al (2011) Proteins encoded in genomic regions associated with immune-mediated disease physically interact and suggest underlying biology. PLoS Genet 7 1:e1001273. 21249183 10.1371/journal.pgen.1001273 PMC3020935

[13] O'Roak BJ, Vives L, Girirajan S, Karakoc E, Krumm N, et al (2012) Sporadic autism exomes reveal a highly interconnected protein network of de novo mutations. Nature 485 7397:246–50. 22495309 10.1038/nature10989 PMC3350576

[14] Sawcer S, Hellenthal G, Pirinen M, Spencer CC, Patsopoulos NA, et al (2011) Genetic risk and a primary role for cell-mediated immune mechanisms in multiple sclerosis. Nature 476 7359:214–9. 21833088 10.1038/nature10251 PMC3182531

[15] Patsopoulos NA, Esposito F, Reischl J, Lehr S, Bauer D, et al (2011) Genome-wide meta-analysis identifies novel multiple sclerosis susceptibility loci. Ann Neurol 70 6:897–912. 22190364 10.1002/ana.22609 PMC3247076

[16] Wang JH, Pappas D, De Jager PL, Pelletier D, de Bakker PI, et al (2011) Modeling the cumulative genetic risk for multiple sclerosis from genome-wide association data. Genome Med 3 1:3. 21244703 10.1186/gm217 PMC3092088

[17] Wu C, Orozco C, Boyer J, Leglise M, Goodale J, et al (2009) BioGPS: an extensible and customizable portal for querying and organizing gene annotation resources. Genome Biol 10 11:R130. 19919682 10.1186/gb-2009-10-11-r130 PMC3091323

[18] Huang da W, Sherman BT, Lempicki RA (2009) Systematic and integrative analysis of large gene lists using DAVID bioinformatics resources. Nat Protoc 4 1:44–57. 19131956 10.1038/nprot.2008.211

[19] Huang da W, Sherman BT, Lempicki RA (2009) Bioinformatics enrichment tools: paths toward the comprehensive functional analysis of large gene lists. Nucleic Acids Res 37 1:1–13. 19033363 10.1093/nar/gkn923 PMC2615629

[20] Kasper LH, Shoemaker J (2010) Multiple sclerosis immunology: The healthy immune system vs the MS immune system. Neurology 74 Suppl 1:S2–8. 20038759 10.1212/WNL.0b013e3181c97c8f

[21] Kerrigan AM, Brown GD (2011) Syk-coupled C-type lectins in immunity. Trends Immunol 32 4:151–6. 21334257 10.1016/j.it.2011.01.002 PMC3074083

[22] Mocsai A, Ruland J, Tybulewicz VL (2010) The SYK tyrosine kinase: a crucial player in diverse biological functions. Nat Rev Immunol 10 6:387–402. 20467426 10.1038/nri2765 PMC4782221

[23] Hesslein DG, Palacios EH, Sun JC, Beilke JN, Watson SR, et al (2011) Differential requirements for CD45 in NK-cell function reveal distinct roles for Syk-family kinases. Blood 117 11:3087–95. 21245479 10.1182/blood-2010-06-292219 PMC3062311

[24] Weinblatt ME, Kavanaugh A, Genovese MC, Musser TK, Grossbard EB, et al (2010) An oral spleen tyrosine kinase (Syk) inhibitor for rheumatoid arthritis. N Engl J Med 363 14:1303–12. 20879879 10.1056/NEJMoa1000500

[25] Yamada T, Sun Q, Zeibecoglou K, Bungre J, North J, et al (1998) IL-3, IL-5, granulocyte-macrophage colony-stimulating factor receptor alpha-subunit, and common beta-subunit expression by peripheral leukocytes and blood dendritic cells. J Allergy Clin Immunol 101 5:677–82. 9600506 10.1016/S0091-6749(98)70177-0

[26] Asquith KL, Ramshaw HS, Hansbro PM, Beagley KW, Lopez AF, et al (2008) The IL-3/IL-5/GM-CSF common receptor plays a pivotal role in the regulation of Th2 immunity and allergic airway inflammation. J Immunol 180 2:1199–206. 18178860 10.4049/jimmunol.180.2.1199

[27] Chen Q, Wang X, O'Neill FA, Walsh D, Fanous A, et al (2008) Association study of CSF2RB with schizophrenia in Irish family and case - control samples. Mol Psychiatry 13 10:930–8. 17667962 10.1038/sj.mp.4002051 PMC4034748

[28] Moskvina V, Craddock N, Holmans P, Nikolov I, Pahwa JS, et al (2009) Gene-wide analyses of genome-wide association data sets: evidence for multiple common risk alleles for schizophrenia and bipolar disorder and for overlap in genetic risk. Mol Psychiatry 14 3:252–60. 19065143 10.1038/mp.2008.133 PMC3970088

[29] Mader S, Lee H, Pause A, Sonenberg N (1995) The translation initiation factor eIF-4E binds to a common motif shared by the translation factor eIF-4 gamma and the translational repressors 4E-binding proteins. Mol Cell Biol 15 9:4990–7. 7651417 10.1128/mcb.15.9.4990 PMC230746

[30] Dowling RJ, Topisirovic I, Alain T, Bidinosti M, Fonseca BD, et al (2010) mTORC1-mediated cell proliferation, but not cell growth, controlled by the 4E-BPs. Science 328 5982:1172–6. 20508131 10.1126/science.1187532 PMC2893390

[31] Colina R, Costa-Mattioli M, Dowling RJ, Jaramillo M, Tai LH, et al (2008) Translational control of the innate immune response through IRF-7. Nature 452 7185:323–8. 18272964 10.1038/nature06730

[32] Hiraoka N, Nakagawa H, Ong E, Akama TO, Fukuda MN, et al (2000) Molecular cloning and expression of two distinct human chondroitin 4-O-sulfotransferases that belong to the HNK-1 sulfotransferase gene family. J Biol Chem 275 26:20188–96. 10781601 10.1074/jbc.M002443200

[33] Mikami T, Mizumoto S, Kago N, Kitagawa H, Sugahara K (2003) Specificities of three distinct human chondroitin/dermatan N-acetylgalactosamine 4-O-sulfotransferases demonstrated using partially desulfated dermatan sulfate as an acceptor: implication of differential roles in dermatan sulfate biosynthesis. J Biol Chem 278 38:36115–27. 12847091 10.1074/jbc.M306044200

